# Combined innate and adaptive immunotherapy overcomes resistance of immunologically cold syngeneic murine neuroblastoma to checkpoint inhibition

**DOI:** 10.1186/s40425-019-0823-6

**Published:** 2019-12-06

**Authors:** Julie Voeller, Amy K. Erbe, Jacob Slowinski, Kayla Rasmussen, Peter M. Carlson, Anna Hoefges, Sabrina VandenHeuvel, Ashley Stuckwisch, Xing Wang, Stephen D. Gillies, Ravi B. Patel, Alvin Farrel, Jo Lynne Rokita, John Maris, Jacquelyn A. Hank, Zachary S. Morris, Alexander L. Rakhmilevich, Paul M. Sondel

**Affiliations:** 1 Department of Pediatrics, University of Wisconsin, 4159 WIMR Bldg., UWCCC, 1111 Highland Ave, Madison, WI 53711 USA; 2 Department of Human Oncology, University of Wisconsin, 4159 WIMR Bldg., UWCCC, 1111 Highland Ave, Madison, WI 53711 USA; 3 Department of Biostatistics and Medical Informatics, University of Wisconsin, Madison, WI USA; 4 Provenance Biopharmaceuticals, Carlisle, MA USA; 5 Children’s Hospital of Philadelphia, Philadelphia, PA USA

**Keywords:** Neuroblastoma, Anti-disialogangliodside (anti-GD2), Immunologically cold tumors, Checkpoint blockade, Radiation, Pediatric cancer, Combination immunotherapy

## Abstract

**Background:**

Unlike some adult cancers, most pediatric cancers are considered immunologically cold and generally less responsive to immunotherapy. While immunotherapy has already been incorporated into standard of care treatment for pediatric patients with high-risk neuroblastoma, overall survival remains poor. In a mouse melanoma model, we found that radiation and tumor-specific immunocytokine generate an in situ vaccination response in syngeneic mice bearing large tumors. Here, we tested whether a novel immunotherapeutic approach utilizing radiation and immunocytokine together with innate immune stimulation could generate a potent antitumor response with immunologic memory against syngeneic murine neuroblastoma.

**Methods:**

Mice bearing disialoganglioside (GD2)-expressing neuroblastoma tumors (either NXS2 or 9464D-GD2) were treated with radiation and immunotherapy (including anti-GD2 immunocytokine with or without anti-CTLA-4, CpG and anti-CD40 monoclonal antibody). Tumor growth, animal survival and immune cell infiltrate were analyzed in the tumor microenvironment in response to various treatment regimens.

**Results:**

NXS2 had a moderate tumor mutation burden (TMB) while N-MYC driven 9464D-GD2 had a low TMB, therefore the latter served as a better model for high-risk neuroblastoma (an immunologically cold tumor). Radiation and immunocytokine induced a potent in situ vaccination response against NXS2 tumors, but not in the 9464D-GD2 tumor model. Addition of checkpoint blockade with anti-CTLA-4 was not effective alone against 9464D-GD2 tumors; inclusion of CpG and anti-CD40 achieved a potent antitumor response with decreased T regulatory cells within the tumors and induction of immunologic memory.

**Conclusions:**

These data suggest that a combined innate and adaptive immunotherapeutic approach can be effective against immunologically cold syngeneic murine neuroblastoma. Further testing is needed to determine how these concepts might translate into development of more effective immunotherapeutic approaches for the treatment of clinically high-risk neuroblastoma.

## Introduction

Neuroblastoma is the most common extracranial solid tumor in pediatrics. Overall survival is poor for high-risk cases and accounts for about 15% of childhood cancer-related mortality [1–3]. About 20% of neuroblastoma tumors are N-MYC amplified, which is a poor prognostic factor [2]. Our prior preclinical work investigating immunotherapy regimens using a tumor-specific monoclonal antibody (mAb) [anti-disialoganglioside (GD2), referred to as “anti-GD2”] together with an immunostimulatory agent [interleukin-2 (IL2)] has already been successfully translated to the clinical setting, which has significantly improved survival for patients with high-risk disease [4]. Current standard of care treatment for patients with high-risk neuroblastoma includes multi-agent chemotherapy, surgical resection, autologous stem cell transplantation, radiation therapy and immunotherapy [with anti-GD2 mAb, granulocyte-macrophage colony-stimulating factor (GM-CSF), IL2 and retinoic acid]. Unfortunately, the rate of progressive and relapsed disease is still high, and some patients do not have a strong enough response to induction and consolidation therapy to be eligible for the subsequent immunotherapy treatment phase. Enhancing the current immunotherapy may play a role in further improving survival for these patients by enabling tumor-selective killing with minimal harm to normal tissues, achieving cures via eradicating all disease sites, generating immune memory and potentially reducing reliance on genotoxic high-dose chemoradiation.

GD2 is a surface antigen that is expressed on tumors of neuroectodermal origin—including neuroblastoma and melanoma—with limited expression in normal tissues, making it a suitable target for antitumor therapy [5–8]. Tumor-reactive mAbs can themselves have direct cytotoxicity and can also enhance the antitumor immune response via antibody-dependent cell-mediated cytotoxicity (ADCC), antibody-dependent cellular phagocytosis and complement-dependent cytotoxicity [9].

We previously demonstrated that local external beam radiation therapy (RT) and intratumoral (IT) injection of the hu14.18-IL2 immunocytokine (IC), a fusion protein linking hu14.18 anti-GD2 mAb and IL2, achieves improved tumor control and survival in mice bearing B78 melanoma, which expresses GD2 [10, 11]. Moreover, the combination of RT and IT injection of IC (IT-IC) triggers an in situ vaccination effect, turning the tumor into a nidus for enhanced antigen recognition by the immune system and generation of a potent adaptive T cell response directed against distant tumors with the help of innate immune cells bearing Fc receptors [10]. This response is augmented by the addition of immune checkpoint blockade with anti-cytotoxic T-lymphocyte-associated protein 4 (anti-CTLA-4), which can deplete T regulatory cells (Tregs) in the tumor microenvironment and enhances antitumor responses in the setting of more advanced disseminated disease [10].

Separately, we showed that a rational combination of innate and adaptive immunotherapeutic approaches can be synergistic, resulting in a potent antitumor effect in syngeneic mice with advanced B78 melanoma [12]. Agonistic anti-CD40 mAb (which activates effector macrophages) and CpG-oligodeoxynucleotides (a toll-like receptor 9 agonist that acts as a danger signal) induce tumor destruction via innate effector cells, leading to increased presentation of tumor antigens and an adaptive T cell response.

Unlike some adult cancers, most pediatric cancers are considered immunologically cold, in that these tumors have a low tumor mutation burden (TMB)—resulting in a lower level of mutation-coded neoantigens—and are associated with limited immune cell infiltrate [13–15]. These cold tumors are more difficult to target with the body’s own adaptive immune system [16–19]. Furthermore, these cold tumors often have fewer antitumor effector immune cells and tend to have more suppressor cells in the tumor microenvironment [20]. Therefore, recent immune checkpoint inhibition that works for some adult cancers does not have the same beneficial effect on these cold pediatric tumors [21–23].

To simulate clinically high-risk disease, we developed an N-MYC driven, low TMB, and high-GD2-expressing syngeneic murine neuroblastoma model by transducing GD2 and GD3 synthase genes into 9464D neuroblastoma cells (referred to as “9464D-GD2”). Separately, we also assessed immunotherapeutic approaches in another neuroblastoma model, NXS2, which expresses GD2 and has a moderate TMB. We expected that RT and IT-IC with or without checkpoint blockade (a regimen that produced a potent in situ vaccination effect in syngeneic mice bearing B78 melanoma tumors) would also be effective against neuroblastoma tumors. Here, we show that RT and IT-IC achieves complete regression with immunologic memory in mice bearing NXS2 tumors, but not in mice bearing the cold 9464D-GD2 tumors. By including additional activation of the innate immune system to enhance immune recognition of the cold 9464D-GD2 tumors, we achieved a markedly improved antitumor effect, as previously reported for murine models of advanced melanoma [12].

## Materials and methods

### Tumor cell lines

The 9464D cell line [obtained from Jon Wigginton, MD, while at the National Cancer Institute (NCI), Bethesda, MD] was derived from spontaneous neuroblastoma tumors arising in TH-MYCN transgenic mice on C57Bl/6 background developed originally by William A. Weiss, MD, PhD (University of California, San Francisco, CA) [24]. To create a high GD2-expressing 9464D cell line (9464D-GD2), as both GD2-synthase and GD3-synthase are required for GD2 presentation on the surface of cells, lentivirus for GD2-synthase and GD3-synthase (pLV-GD2-synthase-puromycin and pLV-GD3-synthase-blastocidin, designed in VectorBuilder) were sequentially transduced into 9464D cells. The 9464D cells were first transduced with GD2-synthase, and positively transduced cells were selected for using 6 μg/ml of puromycin; 9464D-GD2 synthase positive cells were then transduced with GD3-synthase, and positively transduced cells were selected for using 7.5 μg/ml of blasticidin. Stably transduced 9464D + GD2 + GD3+ cells (referred to as “9464D-GD2”) were then single-cell cloned. Two separate 9464D-GD2 clones were used for in vivo experiments.

The NXS2 cell line (kindly obtained from Ralph Reisfeld, PhD, The Scripps Research Institute, La Jolla, CA, and then maintained by Alice Yu, MD, University of California, San Diego, CA) is a moderately immunogenic, highly metastatic, GD2-positive murine neuroblastoma cell line [25]. NXS2 is a hybrid between GD2-negative C1300 (a neuroblastoma tumor that spontaneously arose in A/J mice [26]) and GD2-positive murine dorsal root ganglioma cells (C57Bl/6 J background, but does not express C57Bl/6 H-2 and therefore grows in immunocompetent A/J mice).

Cells were grown in DMEM medium supplemented with 10% FBS, 2 mM L-glutamine, and 100 U/ml penicillin/streptomycin at 37 °C in a humidified 5% CO2 atmosphere. 9464D-GD2 media was also supplemented with 5% M3 base as well as puromycin (6 μg/ml) and blasticidin (7.5 μg/ml) antibiotics to select for cells that retained the GD2 and GD3 synthase genes. GD2 expression and tumor cell viability (> 95%) were verified prior to tumor engraftment. Cells were routinely monitored for *Mycoplasma* by PCR testing as previously described [27].

### Radiation

External beam RT was delivered to in vivo tumors by an X-RAD 320 (Precision X-Ray, Inc., North Branford, CT) in one fraction to a maximum dose of 12 Gy on day 1 of treatment. Mice were immobilized using custom lead jigs that expose the tumor on the dorsal right flank and shield the rest of the mouse.

### Antibodies and Immunocytokine

Hu14.18K322A, a humanized anti-GD2 mAb with a single point mutation K322A, was provided by Children’s GMP, LLC (St. Jude, Memphis, TN) [28]. Hu14.18-IL2 IC was provided by Apeiron Biologics (Vienna, AU) via the NCI (Bethesda, MD) and has been previously described [29]. Each 50 μg dose of IC contains 10 μg IL2 (corresponding to 150,000 IU based on the specific activity determined by the IL-2 sensitive CTLL-2 cell line) fused to 40 μg 14.18 anti-GD2 mAb (based on the molar amounts of IL2 and anti-GD2 mAb in the IC). A once daily IT dose of 50 μg in 0.1 mL IC was administered on days 6 through 10 for all in vivo NXS2 experiments and for 9464D-GD2 experiments when IC was combined with RT alone. For all other 9464D-GD2 experiments, the dose of IT-IC was halved to 25 μg per dose when combined with other immunotherapeutic agents due to concern for significant toxicities observed in preliminary experiments. For example, we observed 5/5 spontaneous deaths in one group by treatment day 9 when 12 Gy was combined with 50 μg IT-IC once daily starting on day 6, 200 μg anti-CTLA-4 on day 6, 50 μg CpG on days 6 and 8, and 500 μg anti-CD40 on day 3. Therefore, 50 μg IT-IC was administered for experiments in Figs. 2 and 3a as this was the standard dose used in previously published studies in combination with RT, while 25 μg IT-IC was administered for experiments in Figs. 3b, 4 and 5 as some mice were treated with IT-IC in combination with other immunotherapeutic agents.

Anti-mouse-CTLA-4 mAb (IgG2c isotype of the 9D9 clone) was provided by Bristol-Myers Squibb (Redwood City, CA) and functions similarly to the IgG2a isotype as previously described [30]. Anti-CTLA-4 mAb was administered intraperitoneally at a dose of 200 μg in 0.2 mL on days 6, 9, and 12. FGK 45.5 hybridoma cells producing the agonistic anti-CD40 antibody were a gift from Fritz Melchers, PhD (Basel Institute for Immunology, Basel, Switzerland). The mAb was obtained from ascites of nude mice injected with the hybridoma cells, and the ascites were then enriched for IgG by ammonium sulfate precipitation. Anti-CD40 mAb was administered at a dose of 500 μg in 0.2 mL intraperitoneally on day 3. CpG-1826 oligodeoxynucleotide (TCCATGACGTTCCTGACGTT) was purchased from TriLink Biotechnologies (San Diego, CA) or Integrated DNA Technologies (Coralville, IA) and administered at a dose of 50 μg in 0.1 mL IT on days 6, 8, and 10. Timing of treatments was selected based on prior studies [10, 12, 31, 32].

### Murine tumor models

Female C57Bl/6 and A/J mice, ages 5 to 7 weeks old, were obtained from Taconic Farms (TAC, Germantown, NY) and from The Jackson Laboratory (JAX, Bar Harbor, ME). Mice were housed in the animal facilities at the Wisconsin Institutes for Medical Research and used in accordance with the Guide for Care and Use of Laboratory Animals. Intradermal tumors were established on the dorsal right flank of mice by injecting 2 × 10^6^ tumor cells in 0.1 mL of PBS using a 30G needle. Syngeneic A/J mice were injected with NXS2 cells and syngeneic C57Bl/6 mice were injected with 9464D-GD2 cells. Perpendicular diameters of the tumor were measured using calipers and tumor volume (mm^3^) was approximated as: (width^2^ x length)/2.

For all in vivo experiments, mice were randomized immediately prior to start of treatment (designated as day 1) into each treatment group by ascending order of tumor size. Approximately half of naïve mice injected with tumor cells were randomized to achieve the needed number of mice with the stated average tumor size when starting treatment. In vivo experiments were performed at least in duplicate with five mice per treatment group, with reproducible results; representative data are shown, except when specifically stated otherwise.

For the NXS2 experiment depicted in Fig. 2, combined data from two replicate experiments are shown (*n* = 7 per treatment group in one experiment and *n* = 5 per treatment group in the second experiment, except for the IT-IC alone group which had four mice). Mice were untreated or treated with 12 Gy alone, IT-IC alone, or 12 Gy and IT-IC.

For the 9464D-GD2 experiment depicted in Fig. 3a, representative data from one experiment are shown for mice treated with 12 Gy alone or 12 Gy and 50 μg IT-IC. For Fig. 3b, mice were randomized to be untreated or treated with 12 Gy alone, anti-CTLA-4 alone, 12 Gy and IT-IC, 12 Gy and anti-CTLA-4, or 12 Gy, IT-IC and anti-CTLA-4. Control treatment groups receiving anti-CTLA-4 alone and RT with anti-CTLA-4 were only performed once, while trends for the remaining treatment groups were replicated in at least duplicate. The experiment in Additional file 1: Figure S1 was performed once with anti-CTLA-4 administered on days 6, 8, and 10, but similar results were previously obtained in the B78 melanoma model (not shown). For the experiments depicted in Fig. 4 and Additional file 2: Figure S2, mice were randomized to be untreated or treated with 12 Gy alone or 12 Gy, IT-IC, anti-CTLA-4, CpG, and anti-CD40.

Tumor-bearing A/J or C57Bl/6 mice rendered tumor-free by combined immunotherapy treatment were rechallenged on day 90 by injecting 2 × 10^6^ NXS2 cells or 1 × 10^6^ 9464D-GD2 cells in 0.1 mL PBS, respectively, into the opposite (left) flank. Aggregate data for 9464D-GD2 rechallenge experiments are shown for mice made tumor-free with aforementioned combinations, which in some mice also included an anti-TEM8 antibody, which is an anti-vascular antibody (kindly provided by Brad St. Croix, PhD, NCI, Bethesda, MD), which did not have a statistically significant effect on our tumor growth curves when combined with the treatment regimen used in these studies (data not shown) [33–36]. Naïve control mice were injected on the left flank with the same number of tumor cells. Mice were sacrificed when tumors exceeded 20 mm in any dimension or if mice demonstrated moribund behavior.

### Flow cytometry

9464D-GD2 tumors were extracted on day 13 and incubated for 30 min at 37 °C in dissociation solution containing HBSS supplemented with 5% FBS, 1 mg/mL collagenase type D, and 100 μg/mL DNase I (Sigma-Aldrich) as previously described [12]. For cell surface staining, cells were incubated with anti-GD2-APC (clone 14G2a; BioLegend), anti-CD45-eF450 (clone 30-F11; eBioscience), anti-CD3-Alexa700 (clone 17A2; BioLegend), anti-CD4-PE-Dazzle594 (clone GK1.5; BioLegend), anti-CD8a-APC-eFluor780 (clone 53–6.7; eBioscience), anti-CD11b-BB700 (clone M1/70; BD Horizon), anti-Ly6G-BV711 (clone 1A8; BioLegend), anti-CD25-BB515 (clone PC61; BD Horizon), anti-FoxP3-PE-Cy7 (clone FJK-16 s; eBioscience), and Ghost Dye Violet 510 (Tonbo Biosciences). Flow cytometry data were acquired using an Attune NxT Flow Cytometer and analyzed using FlowJo version 10.1.

### Immunohistochemistry

To visualize GD2 expression after tumor growth in vivo, immunohistochemistry (IHC) was performed as previously described [10, 11]. Untreated parental 9464D and 9464D-GD2 tumors were excised from 3 mice per group following 8–10 weeks of growth. Additionally, 9464D-GD2 tumors were also excised from 3 mice per group at baseline and 6 and 10 days after RT (12 Gy) to the tumor. Fresh tumor samples were cryo-embedded in OCT solution and sectioned. Frozen sections were fixed in − 20 °C acetone for 10 min and labeled overnight at 4 °C using a 1:200 dilution of anti-GD2-PE (clone 14G2a; BioLegend) and DAPI to stain the nucleus of live cells. Representative images were captured of each tumor specimen at 20x magnification using a Keyence BZ-X800 Fluorescence Microscope or Evos FL 2 Imaging System.

### Cytotoxicity assays

An in vitro ^51^chromium-release cytotoxicity assay was performed as previously described [10, 37]. Parental 9464D and 9464D-GD2 target cells were labeled with ^51^chromium and incubated for 4 h with or without hu14.18K322A and fresh peripheral blood mononuclear effector cells. ADCC was measured using a gamma counter (Packard Cobra II) to quantify release of ^51^chromium.

### Mutational burden analyses

Whole exome sequencing (WES) on the murine models and FASTQ file preparation was performed using the Illumina NextSeq 500 High-Output Flow Cell (read length 2 × 150, 120 Gb, and 400 M reads) by the Sidney Kimmel Cancer Center Cancer Genomics Facility of Thomas Jefferson University (Philadelphia, PA).

The murine models’ WES paired-end FASTQ files were aligned to University of California Santa Cruz’s mouse reference genome mm10 with BWA-MEM (v0.7.17) [38]. Base quality scores were recalibrated using GATK (v4.0.3.0) [39]. Somatic mutations in 9464D and 9464D-GD2, and NXS2 with at least 50x coverage were called with MuTect2 [40] and filtered against A/J, C57BL/6 J, and C57BL/6 T as a panel of normal.

### Statistical analyses

Tumor volume curves are displayed as means ± standard error of mean (SEM) until the first death occurred in the group, except for Fig. 3b where curves are displayed until the second death occurred in the group due to a single incidence of early death during treatment in the anti-CTLA-4 alone group. Tumor growth curves were analyzed using linear mixed-effects models including random intercepts for subjects followed by Tukey’s multiple comparisons adjustment. The tumor volumes were log transformed to account for the log-linear growth pattern. Survival curves were generated using the Kaplan-Meier method and pairwise comparisons were performed using proportional hazards model with a two-way factorial design. An unpaired Student t test on the log-transformed data was performed for the analysis in Fig. 4c. Wilcoxon two sample tests with Benjamini Hochberg adjustment was performed for the analysis in Fig. 5a and percentages are displayed as means ± SEM. All analyses were performed in R 3.5.0. *P* values less than 0.05 were considered significant and are indicated in figures as *** = *P* < 0.001; ** = *P* < 0.01; * = *P* < 0.05; NS = nonsignificant.

## Results

### Development of a syngeneic N-MYC- and GD2-positive cold neuroblastoma murine model

**Fig. 1:**
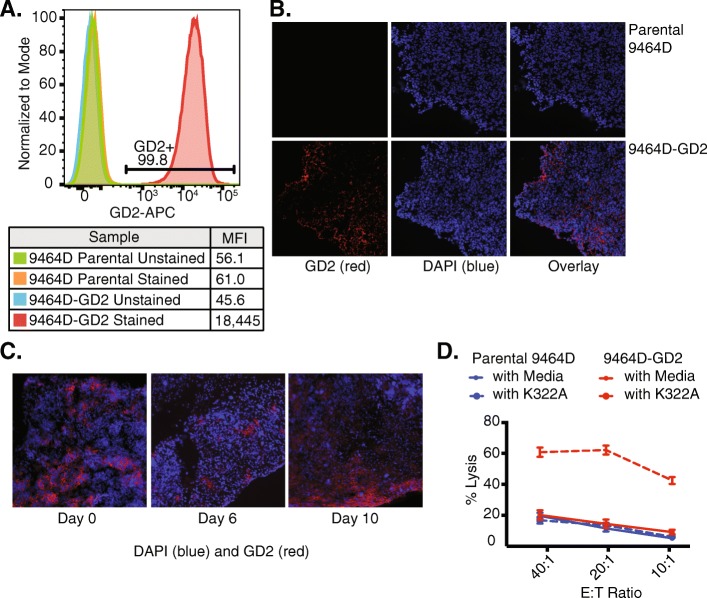
Retained GD2 expression in 9464D-GD2 after growth in vitro and in vivo and increased ADCC. **a** GD2 expression levels in 9464D parental and 9464D-GD2 cells growing in vitro were assessed by flow cytometry. Mean fluorescence intensity (MFI) of GD2 expression is shown for 9464D parental and 9464D-GD2 cells labeled with anti-GD2 mAb compared to the unstained controls. **b** After 8–10 weeks of growth in vivo, 9464D parental (top row) and 9464D-GD2 tumors (bottom row) were harvested and analyzed by IHC for GD2 expression (red, left panel). DAPI was used to stain the nuclei of cells (blue, middle panel), and the overlay of blue and red is in the right panel. **c** 9464D-GD2 tumors were harvested at baseline as well as 6 and 10 days after delivery of 12 Gy to the tumor and analyzed by IHC for GD2 expression. Sections were stained with DAPI alone (blue) anti-GD2-PE (red). **d** A chromium release assay was performed with different effector to target (E:T) ratios to compare cell-mediated cytotoxicity of parental 9464D and 9464D-GD2 cells incubated with or without hu14.18K322A. Percent lysis is shown for each E:T ratio (mean ± SEM)

To simulate clinically high-risk neuroblastoma, we used the syngeneic NXS2 and 9464D murine models. NXS2 is a GD2-expressing hybridoma [41]. While 9464D has been reported to express GD2 in vitro [42], we did not observe expression of GD2 in the 9464D tumor cells by flow cytometry (Fig. 1a). Therefore, we transduced GD2 and GD3 synthase genes into 9464D (referred to as 9464D-GD2). 9464D-GD2 cells have a high level of GD2 expression (Fig. 1a), which was retained after at least 20 passages in vitro (data not shown). Furthermore, GD2 expression was retained in 9464D-GD2 tumors after growth in vivo (Fig. 1b) and was stable at 6 and 10 days after radiation compared to baseline (Fig. 1c). This GD2 expression on the 9464D tumor cells was sufficient to enable ADCC of the cells when incubated with an anti-GD2 mAb (Fig. 1d). As expected, we did not observe a difference in ADCC when the parental 9464D GD2-deficient cells were incubated with or without hu14.18K322A.

### Testing response of syngeneic murine neuroblastomas to RT and IT-IC

**Fig. 2:**
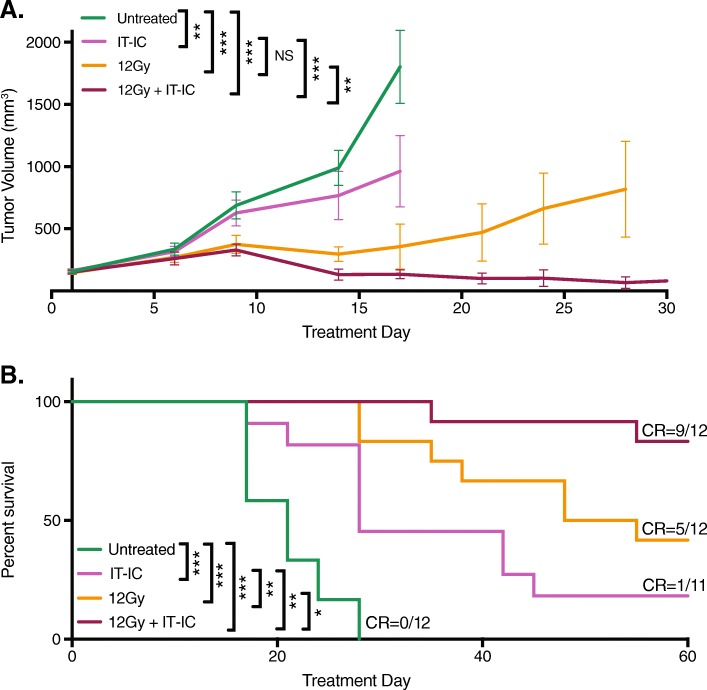
RT and IT-IC produced an in situ vaccination response in mice bearing NXS2 neuroblastoma. Intradermal NXS2 tumors (average starting size of 155 mm^3^ on day 15 post tumor cell implantation) were untreated or treated with IT-IC alone, 12 Gy alone, or 12 Gy and IT-IC. Tumor growth (**a**) and survival (**b**, *p* values are indicated in the table) curves are shown for each treatment group, with disease-free mice at day 60 denoted as complete responses (CR)

To investigate whether an in situ vaccination response could be induced in syngeneic A/J mice bearing an NXS2 neuroblastoma (average tumor size 155 mm^3^ at the start of treatment), we measured tumor growth following treatment with 12 Gy alone, IT-IC alone, 12 Gy and IT-IC, or no treatment (Fig. 2). For those animals treated with RT and IT-IC, we observed complete tumor regression in 42% (5/12) of animals by day 30 (Fig. 2a), with 83% (10/12) surviving past 60 days and 75% (9/12) exhibiting disease-free survival past 60 days (Fig. 2b). For those animals treated with RT alone, 17% (2/12) had complete tumor regression by day 30 and 42% (5/12) survived past 60 days. For those animals treated with IT-IC alone, 27% (3/11) had complete tumor regression by day 30, but only one of these three had tumor-free survival past 60 days while one died spontaneously and one had tumor regrowth by day 46. None of the control untreated mice survived past 30 days (Fig. 2a). In summary, while there was no significant difference in tumor growth for those mice treated with RT alone versus IT-IC alone, mice that were treated with a combination of RT and IT-IC had a significant slowing of tumor growth and improved survival compared to all other groups, with the majority of mice remaining tumor-free past 90 days.

The 9 mice rendered disease-free by their treatment with RT and IT-IC (Fig. 2b) were rechallenged with NXS2 tumors; 89% (8/9) rejected the NXS2 rechallenge. In contrast, all 10/10 naïve mice challenged with NXS2 in parallel showed progressive NXS2 growth, suggesting that the treated mice were cured and developed an immune memory response. These results are aligned with our previously published studies showing that RT and IT-IC induces an in situ vaccination response in syngeneic mice bearing B78 melanoma, and substantially improves upon tumor responses and animal survival compared to treatment of mice bearing NXS2 with RT and anti-GD2 mAb [10, 11].

Genomic analyses comparing NXS2 and 9464D-GD2 tumor cells confirmed that the latter is N-MYC mutated and had a lower tumor mutation burden (Table 1). We used this syngeneic 9464D-GD2 model in immune competent mice to simulate an immunologically cold, N-MYC-driven, high-risk clinical neuroblastoma.

**Table 1:** Mutational burden in NXS2 vs 9464D-GD2 cells. Genomic analysis of tumor cell lines reveals that the 9464D and 9464D-GD2 neuroblastoma cell lines have lower mutation burdens compared to the NXS2 neuroblastoma cell line. All 3 lines show TP53 mutations

Tumor	TMB (Mutations/Megabase)	TP53 Mutations	
		Mouse	Human Homolog
NXS2	13.9	V167 L	V173 L
Parental 9464D	1.5	A132P	A138P
9464D-GD2	1.6	A132P	A138P

**Fig. 3:**
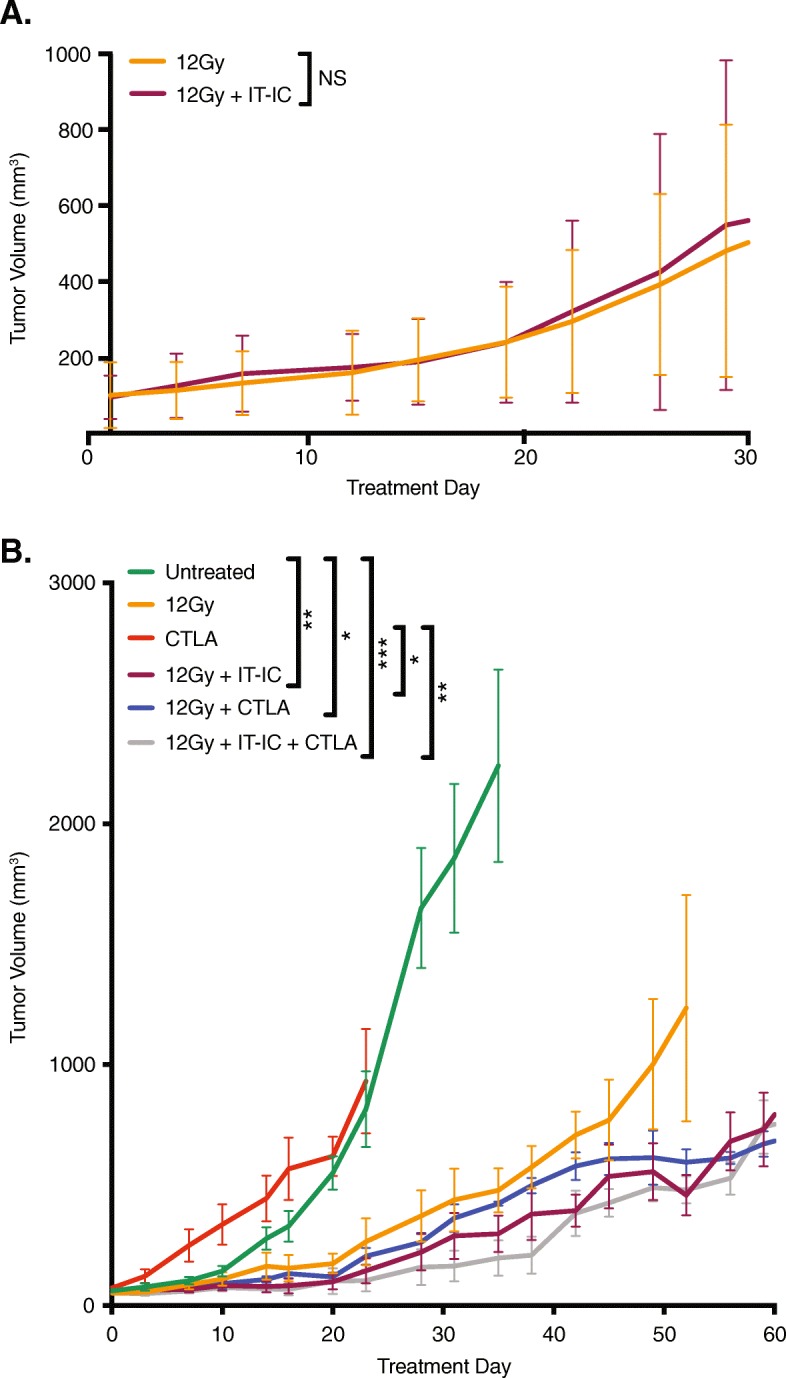
RT and IT-IC does not produce an in situ vaccination effect in immunologically cold 9464D-GD2 neuroblastoma, and response is not improved with immune checkpoint inhibition. Tumor growth curves are shown for intradermal 9464D-GD2 tumors treated with RT alone or RT and IT-IC (**a**) as well as tumors treated with the addition of checkpoint blockade with anti-CTLA-4 (CTLA) to RT with or without ½ dose IT-IC (**b**, *p* values are indicated in Additional file 3: Table S1)

Using the immunologically cold 9464D-GD2 tumor, we next investigated whether this same in situ vaccination response would be induced via RT and IT-IC treatment (Fig. 3a). We have previously observed in several tumor models that larger tumor volumes at the start of treatment attenuates the antitumor response to this form of immunotherapy treatment [25]; we again observed this phenomenon in our NXS2 and 9464D-GD2 models presented here (data not shown). However, even with a smaller average starting volume of 100 mm^3^, none of the mice in the RT and IT-IC group achieved a complete tumor regression, and in this experiment, the addition of IT-IC to RT did not appear to slow 9464D-GD2 tumor growth compared to the RT alone.

We previously observed that the addition of anti-CTLA-4 to RT and IT-IC was effective against mice bearing a single large primary or two B78 melanoma tumors [10]. For mice bearing 9464D-GD2 tumors, there was no significant difference in tumor growth of mice treated with anti-CTLA-4 alone compared to untreated tumors (Fig. 3b). In contrast, there was slight (nonsignificant) slowing of tumor growth when mice were treated with RT alone compared to untreated mice. However, combining RT and IT-IC, RT and anti-CTLA-4, or RT, IT-IC and anti-CTLA-4 did not cause any significant tumor growth inhibition over that seen with RT alone. Furthermore, while RT and combined therapies with IT-IC and/or anti-CTLA-4 had significant slowing of tumor growth compared to untreated tumors, none of the mice in any of the treatment groups achieved a complete response. These results are consistent with our hypothesis that cold tumors, such as N-MYC driven 9464D-GD2, are less responsive to combination immunotherapy, including the addition of an RT and IT-IC in situ vaccination regimen to checkpoint blockade.

### A combined innate and adaptive immunotherapeutic approach is effective against cold N-MYC neuroblastoma

**Fig. 4:**
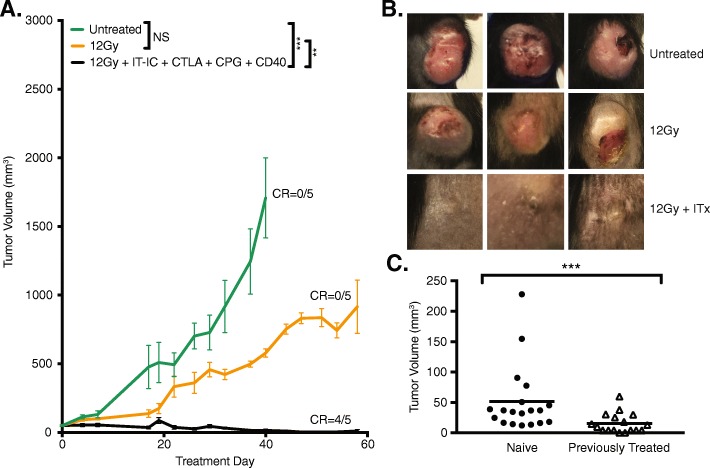
A combined innate and adaptive immunotherapeutic approach leads to 9464D-GD2 tumor regression and immunological memory. **a** Tumor growth curves are shown for TAC mice bearing intradermal 9464D-GD2 tumors (about 50mm^3^) that were untreated or treated with RT alone or RT and combined ½ dose IT-IC, anti-CTLA-4 (CTLA), CpG and anti-CD40 (CD40). Tumor-free mice on day 60 are denoted as number of CR of total mice in the group. **b**) Photographs of 3 representative TAC mice per group taken on day 24 show contrasting tumor size and appearance after 12 Gy alone or 12 Gy and immunotherapy (ITx, or combined ½ dose IT-IC, anti-CTLA-4, CpG, and anti-CD40) compared to untreated control mice. **c** Mice previously bearing a 9464D-GD2 tumor on the right flank that had complete response to treatment were rechallenged on day 90 by injecting 9464D-GD2 cells into the left flank. Tumor volumes on day 30 after tumor cell injection are significantly larger for naïve mice compared to previously treated mice (*p* = 0.0003)

Our next step was to enhance the response of cold neuroblastoma tumors to immunotherapy. Based on previous observations in mice bearing advanced B78 melanoma [12], we hypothesized that a combination of innate and adaptive immunotherapeutic approaches would increase the antitumor efficacy against 9464D-GD2 neuroblastoma. Accordingly, in addition to RT, ½ dose IT-IC and anti-CTLA-4, we included treatment with CpG and anti-CD40. We observed significantly improved tumor control with this combined regimen, with 4 of 5 mice (80%) achieving complete tumor regression (Fig. 4a). By day 24, untreated control tumors were significantly larger in size, nodular and sometimes ulcerated, while tumors treated with combined innate and adaptive immunotherapy—that is, 12 Gy and combined ½ dose IT-IC, anti-CTLA-4, CpG, and anti-CD40—were significantly smaller and appeared mostly scarred by day 24 (Fig. 4b). Similar antitumor responses to those shown in Fig. 4a in mice bearing 9464D-GD2 tumors were also seen with RT combined with IT-IC, anti-CTLA-4, CpG, and anti-CD40 when we tested certain dose-related modifications, i.e., 50 μg IT-IC and 250 μg anti-CD40 compared to 25 μg IT-IC and 500 μg anti-CD40 (data not shown). In both the B78 melanoma model (data not shown) and the 9464D-GD2 model (Additional file 1: Figure S1), full combined treatment with RT, IT-IC, anti-CTLA-4, and anti-CD40/CpG was more effective than different double and triple combinations of these agents (with anti-CD40 and CpG being considered as one synergistic treatment activating innate immunity), where in both tumor models only full combined treatment resulted either in complete tumor regression in some mice or in the strongest tumor growth suppression.

Silvan and colleagues recently showed that in some tumor models commensal microbiota, specifically *Bifidobacterium* typically found in the gut of C57Bl/6 mice obtained from JAX (but not from TAC), can play a significant role in slowing tumor growth [43]. They further showed that the antitumor response is due to the regulation of antitumor immunity and augmented by treatment with anti-programmed cell death protein 1 ligand 1 mAb (anti-PD-L1)—an effect that was mediated by enhanced CD8+ T cell priming and accumulation in the tumor microenvironment [43]. Based on these data, we aimed to determine if different mouse strains could influence response to innate and adaptive immunotherapy approaches in mice bearing 9464D-GD2 tumors. In contrast to previously published findings with other tumor models, we did not observe a significant difference in tumor growth in JAX mice (Additional file 2: Figure S2) compared to TAC mice (Fig. 4a) of untreated 9464D-GD2 tumors or tumors treated with RT alone or RT and combined ½ dose IT-IC, anti-CTLA-4, CpG, and anti-CD40.

### Evidence for antitumor memory

To determine whether a memory response was generated by the RT and combined ½ dose IT-IC, anti-CTLA-4, CpG, and anti-CD40 regimen, we rechallenged mice that achieved complete regression of their initial 9464D-GD2 tumor with the same tumor cells on the opposite flank on day 90 (Fig. 4c). Tumors engrafted in all naïve mice (19/19). Even though the majority of previously treated mice (15/17, or 88%) did not reject rechallenge, there was significant slowing of tumor growth in previously treated mice compared to naïve mice. The average tumor volume on day 30 after tumor cell injection (after which tumor engraftment becomes evident in naïve mice) in previously treated mice (15.4 mm^3^) was significantly smaller than that of naïve mice (51.5 mm^3^, *p* = 0.012), suggesting the presence of a memory response.

### Phenotype of tumor infiltrating immune cells in mice treated with RT and combined IT-IC, anti-CTLA-4, CpG, and anti-CD40

**Fig. 5:**
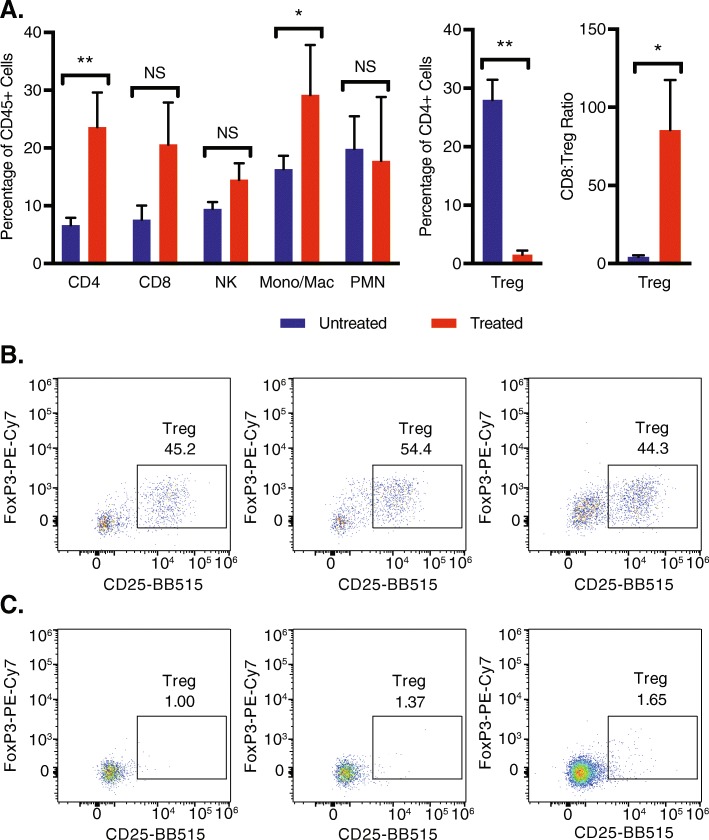
Treated 9464D-GD2 tumors have significantly fewer T regulatory cells, with a higher CD8+ T cell to Treg ratio, and more CD4+ T cells and monocytes/macrophages compared to untreated tumors. Untreated 9464D-GD2 tumors and tumors treated with 12 Gy and combined ½ dose IT-IC, anti-CTLA-4 (CTLA), CpG, and anti-CD40 (CD40) were harvested on treatment day 13, and tumor microenvironment was analyzed by flow cytometry (**a**). Representative dot plots of Treg populations (defined as CD25 + FoxP3+ of CD45 + CD4+ live cells) are shown for three representative untreated (**b**) and treated (**c**) tumors (numerical values shown are the % of CD45 + CD4+ live cells that are Tregs)

Analysis of cells in the 9464D-GD2 tumor microenvironment on treatment day 13 revealed an increase of CD4+ T cells, monocytes (Mono)/macrophages (Mac), CD8 to Treg ratio, and reduction of Tregs, whereas the percentages of NK cells and neutrophils were unchanged (Fig. 5). The significant reduction in Tregs seen here after treatment of 9464D-GD2 with RT combined with ½ dose IT-IC, anti-CTLA-4, CpG, and anti-CD40 was also seen after treatment of B78 melanoma with IT-IC, anti-CTLA-4, CpG, and anti-CD40 without radiation [12], suggesting that this immunotherapy plays a significant role in reducing Tregs in the tumor microenvironment.

## Discussion

Immune checkpoint inhibitors, including anti-CTLA-4 and anti-PD-1 mAbs, have recently been shown to be effective in treating some adult cancers. This has generated tremendous momentum to incorporating mechanisms to “release the brakes” on the immune system to fight cancer [44]. However, many pediatric cancers are considered immunologically cold—that is, they have a low mutation burden and low neoantigen load, as well as fewer tumor infiltrating lymphocytes—and do not typically respond to treatment with checkpoint inhibition alone [21–23]. We previously showed that IT-IC, with or without RT and anti-CTLA-4, can serve as an in situ vaccination, enhancing local antitumor effects and generating a systemic adaptive T cell response against distant tumors [10]. We have further demonstrated that a combination of immunotherapeutic approaches targeting innate and adaptive immunity has a synergistic antitumor effect against well-established tumors in a syngeneic B78 melanoma murine model; specifically, we showed that adding CpG and anti-CD40 to IT-IC and anti-CTLA-4 provided substantially greater antitumor efficacy than CpG and anti-CD40 alone or IT-IC and anti-CTLA-4 alone [12]. Anti-CD40/CpG activate innate immunity, mainly macrophages, while anti-CTLA-4 releases the brakes on effector T cells and can deplete CD4+ Tregs in the tumor microenvironment.

Here we also show that same RT and IT-IC regimen that we have previously shown to be effective against the B78 melanoma induced an in situ vaccination response with complete tumor regression and immunologic memory in the NXS2 neuroblastoma model, an improvement upon what we have previously seen with RT and anti-GD2 mAb in this model [10]. In contrast, even with the addition of anti-CTLA-4, this regimen was not effective against 9464D-GD2 neuroblastoma, which has a lower mutational burden and is more immunologically cold. Instead, addition of a combined innate and adaptive immunotherapeutic approach with RT and combined IT-IC, anti-CTLA-4, CpG and anti-CD40 was effective against this cold 9464D-GD2 tumor, with some mice achieving complete tumor regression. The kinetics of tumor growth after rechallenge were slowed in these tumor-free mice compared to naïve mice, suggesting the presence of a memory response. We hypothesize that because these 9464D-GD2 tumor cells have relatively low MHC class I expression (data not shown), immune memory could be better detected by using a variant of this tumor expressing high MHC class I for rechallenge. This hypothesis will be tested in future studies.

In certain settings, differences in the gut microbiome affect response to cancer treatment with chemotherapy and immunotherapy [45–48]. In contrast to a prior study evaluating other tumor models that show more robust antitumor responses to immunotherapy among mice obtained from specific vendors [43], we did not observe slowed tumor growth or improved antitumor immunotherapeutic responses in C57Bl/6 mice obtained from Jackson (JAX) compared to C57Bl/6 mice obtained from Taconic (TAC) bearing the immunologically cold, syngeneic 9464D-GD2 tumors in the conditions tested here. While differences in the microbiome may play a role in modulating the response of 9464D-GD2 tumors to immunotherapy, we were not able to detect a difference in the responses tested here between JAX and TAC C57Bl/6 mice. Alternatively, it is possible that differences in the gut microbiome may be less relevant for cold tumor models with poor immunogenicity. This question warrants further investigation.

It is clear that immunologically cold tumors are less responsive to treatment with individual immunotherapeutic agents alone—such as checkpoint blockade—or even with combined agents—such as RT and IT-IC—which have been potent against more immunogenic tumors like NXS2 neuroblastoma and B78 melanoma. In combination, however, immunotherapeutic agents and other regimens can be synergistic and generate a potent antitumor response by increasing tumor antigen presentation and turning on immune recognition to neoantigens or tumor selective endogenous, germ-line controlled self-proteins. Our results show that this improved response of immunologically cold 9464D-GD2 to RT and combined IT-IC, anti-CTLA-4, CpG, and anti-CD40 is associated with increased CD4+ T cell infiltration and decreased presence of Tregs within the tumor microenvironment. The roles of CD4 and CD8 T cells and macrophages in this model will be further investigated in future studies.

Future studies can further investigate the evolution of immune cell infiltrate in these cold tumors over time after treatment as well as an approach to treating mice bearing metastatic cold tumors or spontaneously arising neuroblastoma such as in the TH-MYCN transgenic mouse model [24, 49] The potential toxicities of this combined regimen must also be carefully considered, particularly when considering translation to the clinical setting—for example, we presume that treatment-related deaths were observed when anti-CD40 and IL2 were combined at full doses due to cytokine-release storm. It will be important to determine if this toxicity can be overcome, without losing efficacy, by replacing systemic anti-CD40 injection with the IT injection of a smaller dose of anti-CD40, as we showed in a different study [50]. The role of radiation must also be carefully studied to optimally enhance immune activation in the setting of a radiosensitive tumor such as neuroblastoma. A recent study found that radiation given in repeated doses below the dose threshold for induction of DNA exonuclease Trex1 can optimally stimulate antitumor effector cells and enhance response to immunotherapy [51]. It remains to be determined whether and how such dose thresholds may correlate with the intrinsic radiosensitivity of a tumor, and further preclinical studies may help elucidate this interaction.

## Conclusions

We show here that a combined innate and adaptive immunotherapeutic regimen can achieve potent antitumor killing and long-lasting immunologic memory in a cold neuroblastoma model. These preclinical data will inform clinical investigations of how immunotherapy can further enhance current standard of care treatment approaches combining radiation and tumor-specific immunotherapy for patients with high-risk neuroblastoma. Incorporation of novel immunotherapeutic approaches has the potential to not only improve survival of this patient population, but also reduce reliance on genotoxic high-dose chemoradiation.

## Supplementary information

**Additional file 1: Figure S1.** Antitumor effect of RT, IC, anti-CTLA-4, and anti-CD40/CpG against 9464D-GD2 neuroblastoma. Tumor growth curves are shown for mice bearing intradermal 9464D-GD2 tumors (about 50mm^3^) that were untreated or treated with RT alone, or RT and IT-IC and anti-CTLA-4, or RT and anti-CD40/CpG, or IT-IC and anti-CTLA-4 and anti-CD40/CpG, or RT and combined IT-IC, anti-CTLA-4, and anti-CD40/CpG. Tumor-free mice on day 30 (if any) are denoted as number of CR of total mice in the group. Combined treatment with RT, IT-IC, anti-CTLA-4, and anti-CD40/CpG resulted in complete tumor regression in one mouse and in the strongest tumor growth suppression in other mice.

**Additional file 2: Figure S2.** Response of immunologically cold, syngeneic 9464D-GD2 tumors in C57Bl/6 mice obtained from Jackson (JAX) compared to Taconic (TAC). Tumor growth curves are shown for JAX mice bearing intradermal 9464D-GD2 tumors (about 50mm^3^) that were untreated or treated with RT alone or RT and combined IT-IC, anti-CTLA-4, CpG and anti-CD40. Tumor-free mice on day 60 are denoted as number of CR of total mice in the group. For each treatment group tested in TAC mice (shown in Fig. 4a) and JAX mice (shown here) in the same experiment, there was no significant difference in tumor response between TAC versus JAX mice.

**Additional file 3: Table S1.** *P* values of pairwise comparisons of 9464D-GD2 tumor growth curves after treatment corresponding to Fig. 3b. *P* values of pairwise comparisons of tumor growth curves of untreated intradermal 9464D-GD2 tumors and tumors treated with RT alone, anti-CTLA-4 (CTLA) alone, RT and IT-IC, RT and anti-CTLA-4, or RT and IT-IC and anti-CTLA-4.

## Data Availability

The datasets generated and/or analyzed during the current study are not publicly available due to their relevance only for the experiments presented here but are available from the corresponding author on reasonable request.

## Abbreviations

ADCC: Antibody-dependent cell-mediated cytotoxicity
anti-CTLA-4 or CTLA: Anti-cytotoxic T-lymphocyte-associated protein 4
anti-PD-L1: Anti-programmed cell death protein 1 ligand 1 mAb
CR: Complete response
GD2: Disialoganglioside
IC: Immunocytokine
IL2: Interleukin-2
IT: Intratumoral
JAX: The Jackson Laboratory
mAb: Monoclonal antibody
NK: Natural killer
RT: Radiation therapy
SEM: Standard error of mean
TAC: Taconic Farms
TMB: Tumor mutation burden
Tregs: T regulatory cells
WES: Whole exome sequencing
