# Supplementary figures and images for: Combined innate and adaptive immunotherapy overcomes resistance of immunologically cold syngeneic murine neuroblastoma to checkpoint inhibition
Source: J Immunother Cancer. 2019 Dec 6;7:344. doi: 10.1186/s40425-019-0823-6 (PMC6898936; doi:10.1186/s40425-019-0823-6)

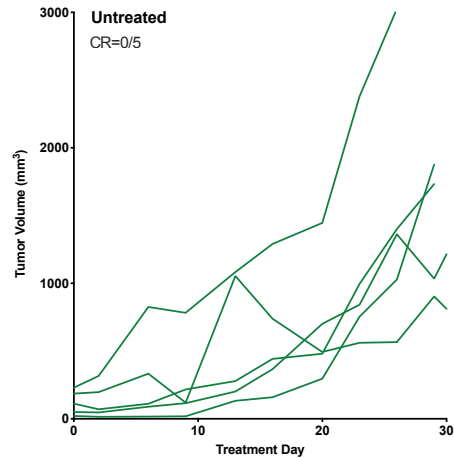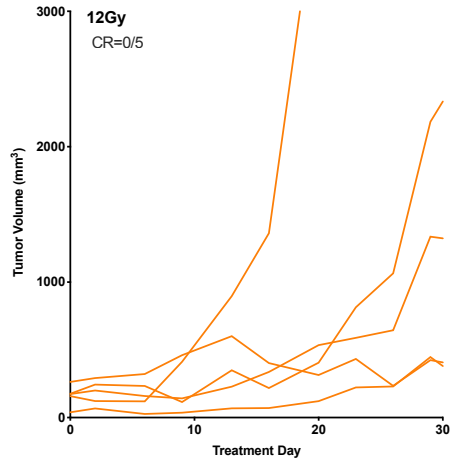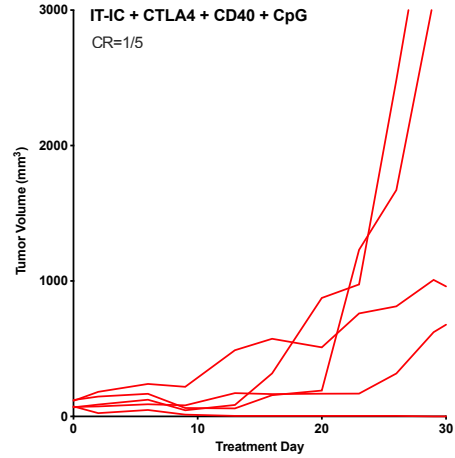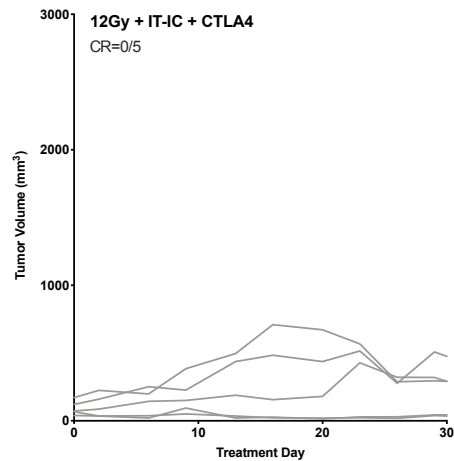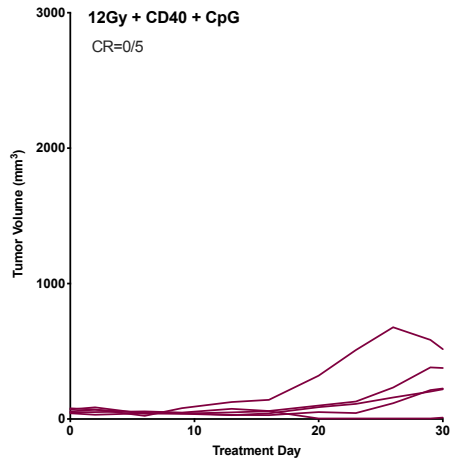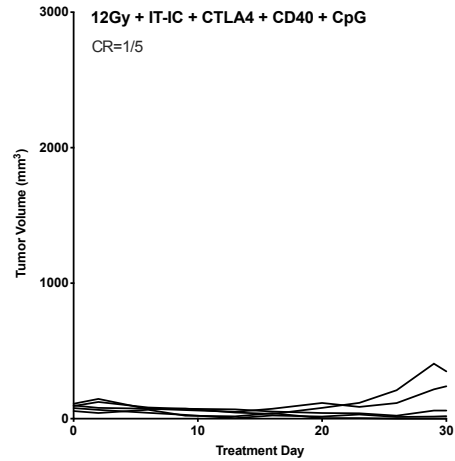

Supplement: Supplementary file 1 — Additional file 1: Figure S1. Antitumor effect of RT, IC, anti-CTLA-4, and anti-CD40/CpG against 9464D-GD2 neuroblastoma. Tumor growth curves are shown for mice bearing intradermal 9464D-GD2 tumors (about 50mm3) that were untreated or treated with RT alone, or RT and IT-IC and anti-CTLA-4, or RT and anti-CD40/CpG, or IT-IC and anti-CTLA-4 and anti-CD40/CpG, or RT and combined IT-IC, anti-CTLA-4, and anti-CD40/CpG. Tumor-free mice on day 30 (if any) are denoted as number of CR of total mice in the group. Combined treatment with RT, IT-IC, anti-CTLA-4, and anti-CD40/CpG resulted in complete tumor regression in one mouse and in the strongest tumor growth suppression in other mice. [file 40425_2019_823_MOESM1_ESM.pdf]

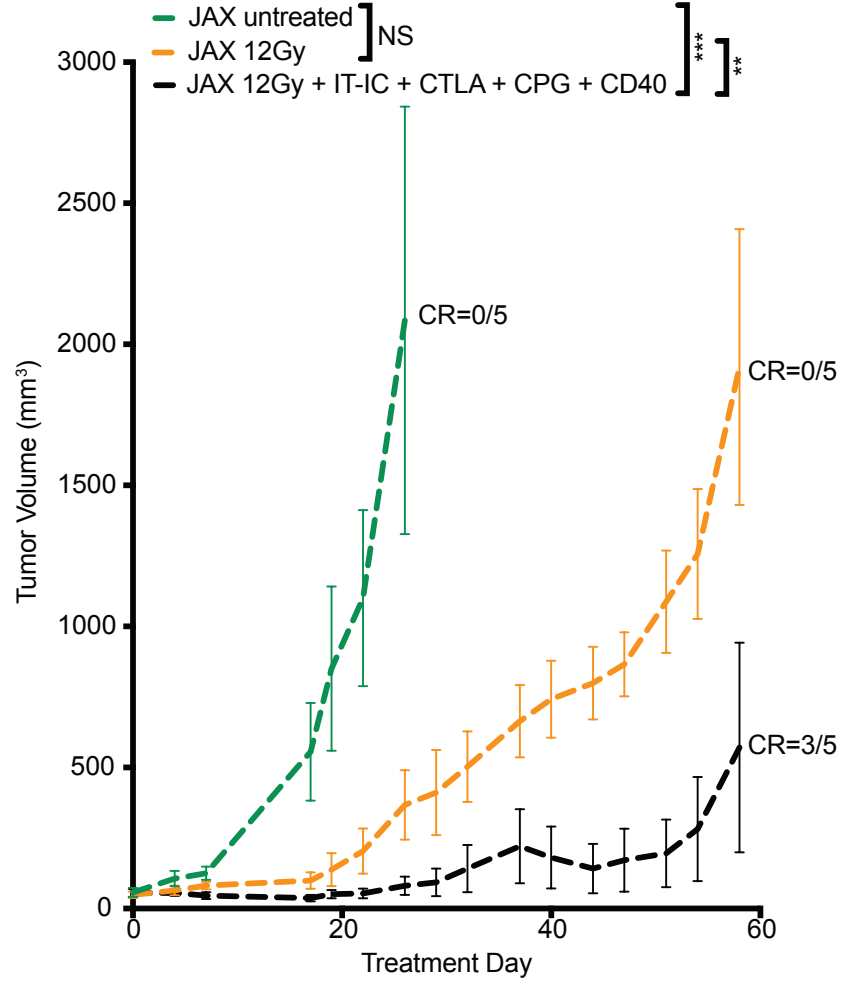

Supplement: Supplementary file 2 — Additional file 2: Figure S2. Response of immunologically cold, syngeneic 9464D-GD2 tumors in C57Bl/6 mice obtained from Jackson (JAX) compared to Taconic (TAC). Tumor growth curves are shown for JAX mice bearing intradermal 9464D-GD2 tumors (about 50mm3) that were untreated or treated with RT alone or RT and combined IT-IC, anti-CTLA-4, CpG and anti-CD40. Tumor-free mice on day 60 are denoted as number of CR of total mice in the group. For each treatment group tested in TAC mice (shown in Fig. 4a) and JAX mice (shown here) in the same experiment, there was no significant difference in tumor response between TAC versus JAX mice. [file 40425_2019_823_MOESM2_ESM.pdf]
