# Supplementary material for: Combined innate and adaptive immunotherapy overcomes resistance of immunologically cold syngeneic murine neuroblastoma to checkpoint inhibition
Source: J Immunother Cancer. 2019 Dec 6;7:344. doi: 10.1186/s40425-019-0823-6 (PMC6898936; doi:10.1186/s40425-019-0823-6)
Supplement: Supplementary file 3 — Additional file 3: Table S1. P values of pairwise comparisons of 9464D-GD2 tumor growth curves after treatment corresponding to Fig. 3b. P values of pairwise comparisons of tumor growth curves of untreated intradermal 9464D-GD2 tumors and tumors treated with RT alone, anti-CTLA-4 (CTLA) alone, RT and IT-IC, RT and anti-CTLA-4, or RT and IT-IC and anti-CTLA-4. [file 40425_2019_823_MOESM3_ESM.docx]

|  | **12Gy** | **CTLA** | **12Gy + IT-IC** | **12Gy + CTLA** | **12Gy + IT-IC + CTLA** |
| --- | --- | --- | --- | --- | --- |
| **Untreated** | 0.1302 | 0.9976 | **0.0045** | **0.0318** | **0.0006** |
| **12Gy** | N/A | 0.2815 | 0.6111 | 0.9754 | 0.1738 |
| **CTLA** | N/A | N/A | **0.0125** | 0.0801 | **0.0017** |
| **12Gy + IT-IC** | N/A | N/A | N/A | 0.9571 | 0.9476 |
| **12Gy + CTLA** | N/A | N/A | N/A | N/A | 0.5411 |

**Table S1.** ***P* values of pairwise comparisons of 9464D-GD2 tumor growth curves after treatment corresponding to Figure 3B.** *P* values of pairwise comparisons of tumor growth curves of untreated intradermal 9464D-GD2 tumors and tumors treated with RT alone, anti-CTLA-4 (CTLA) alone, RT and IT-IC, RT and anti-CTLA-4, or RT and IT-IC and anti-CTLA-4.
